# Bpifcl modulates *kiss2* expression under the influence of 11-ketotestosterone in female zebrafish

**DOI:** 10.1038/s41598-017-08248-8

**Published:** 2017-08-11

**Authors:** Shogo Moriya, Nabila Tahsin, Ishwar S. Parhar

**Affiliations:** Brain Research Institutes, Monash University Malaysia, Jalan Lagoon Selatan, Bandar Sunway, Selangor, 47500 Malaysia

## Abstract

The bactericidal/permeability-increasing (BPI) fold-containing (BPIF) superfamily of genes expressed in the brain are purportedly involved in modulating brain function in response to stress, such as inflammation. Kisspeptin, encoded by *kiss*, is affected by inflammation in the brain; therefore, BPIF family genes might be involved in the modulation of kisspeptin in the brain. In this study, we investigated the expression of BPIF family C, like (*bpifcl*) in zebrafish brain and its involvement in *kiss2* regulation. The identified, full-length sequence of a *bpifcl* isoform expressed in the zebrafish brain contained the BPI fold shared by BPIF family members. *bpifcl* mRNA expression in female zebrafish brains was significantly higher than that in males. Exposure of female zebrafish to 11-ketotestosterone decreased *bpifcl* and *kiss2* mRNA expression. *bpifcl* knockdown by *bpifcl*-specific small interfering RNA administration to female zebrafish brain decreased *kiss2* mRNA expression. *bpifcl* expression was widely distributed in the brain, including in the dorsal zone of the periventricular hypothalamus (Hd). Furthermore, *bpifcl* was also expressed in KISS2 neurons in the Hd. These results suggest that the Bpifcl modulates *kiss2* mRNA expression under the influence of testosterone in the Hd of female zebrafish.

## Introduction

The bactericidal/permeability-increasing (BPI) fold-containing (BPIF) superfamily of genes is functionally classified into five groups: palate, lung and nasal epithelium clone (PLUNC); lipopolysaccharide-binding protein (LBP); BPI protein; phospholipid transfer protein (PLTP); cholesterol ester transfer protein (CETP)^1, 2^. All BPIF family members share the BPI fold, which has an elongated, boomerang shape consisting of two distinct N- and C-terminal domain barrels with a highly similar secondary structure^3^. Both domains consist of two twisted, anti-parallel β-sheets and two α-helices, with both domains connected to each other by a β-sheet^4^.

BPIF family of genes are expressed in a wide range of vertebrates^5^ and mainly involved in the innate immune system and lipoprotein metabolism. BPI and LBP are plasma proteins that play an important role in the innate immune system against invading pathogens owing to a high affinity for the lipopolysaccharides of Gram-negative bacteria^3, 6^. PLUNC proteins are suggested to be host defence molecules because their expression alters in response to inflammation caused by smoking, chemical irritants, and infection^7, 8^. CETP and PLTP are both plasma proteins that play an important role in lipoprotein metabolism^9, 10^. Recently, the expression of BPIF family of genes has been reported in the brain^11^. We have also reported the expression of BPIF family of genes, such as *bpifa1*, *gm1006* and *rya3* in the mouse brain, particularly in the preoptic area^12^. The expression of these genes decreases with age. Because ageing causes oxidative stress and inflammation in the brain^13^, the identified BPIF family of genes might respond to oxidative stress and inflammation similar to the PLUNC protein group in the brain.

Kisspeptin is encoded by *kiss*, expressed in various vertebrates^14^, and involved in various physiologies in the brain, such as reproduction^15^, memory^16^ and metabolism^17^. Kisspeptin also interacts with the adrenergic, serotonergic and GABAergic systems^16^; therefore, kisspeptin is a key modulator of brain function. Kisspeptin is a molecule known to be affected by inflammation in the brain^18, 19^. Since BPIF family genes, particularly BPI, LBP and PLUNC groups, respond to inflammatory agents, BPIF family of genes may interact with kisspeptin to modulate brain functions. Because the regulatory mechanisms of kisspeptin have not been fully identified, the BPIF family-kisspeptin interaction could be a novel kisspeptin regulatory mechanism in the brain.

In zebrafish, three BPIF family of genes, *pltp* (ENSDARG00000104495), *cetp* (ENSDARG00000030872) and BPIF family C, like (*bpifcl*; ENSDARG00000099980) are predicted. The zebrafish CETP protein has similar functionality to human CETP^20^, while zebrafish PLTP is annotated as part of the PLTP group owing to its sequence similarity with mammals^21^. Meanwhile, *bpifcl* has not been classified into a sub-group of the BPIF family and has unknown function. *bpifcl* is located on chromosome 9 and predicted to have three isoforms. In the current study, we investigated the involvement of *bpifcl* in the regulation of *kiss2* in the zebrafish brain. *bpifcl* isoforms expressed and localised in zebrafish brain were initially identified. Correlations between *bpifcl* and *kiss2* mRNA expression were investigated by comparing males and females and different developmental stages. Furthermore, the effect of *bpifcl* knockdown was evaluated to investigate the involvement of *bpifcl* in the regulation of *kiss2* expression.

## Results

### Full-length *bpifcl* mRNA sequence

The identified *bpifcl* isoform (Genbank accession no. KX459407) consisted of 1974 nucleotides spanning 15 exons when compared to the published zebrafish genome. The coding region starts at the 65th base of exon 1 and extends to the 57th base of exon 15. Only this isoform was identified in the current study. The coding sequence was identical to the predicted *bpifcl* (Accession no. ENSDARG00000099980). Simple Modular Architecture Research Tool (SMART) prediction indicated that the N- and C-terminal domains of BPIF family proteins consisted of amino acids (aa) 24-243 and 258-461, respectively (Supplementary Fig. 1A). Phylogenetic tree analysis revealed that the estimated aa sequence of Bpifcl is closer to Pltp than Cetp (Supplementary Fig. 1B). The aa identity between zebrafish Bpifcl and human BPIFC (ENSG00000184459) was 19.5%.

### Localisation of *bpifcl* in zebrafish brain

**Figure 1:**
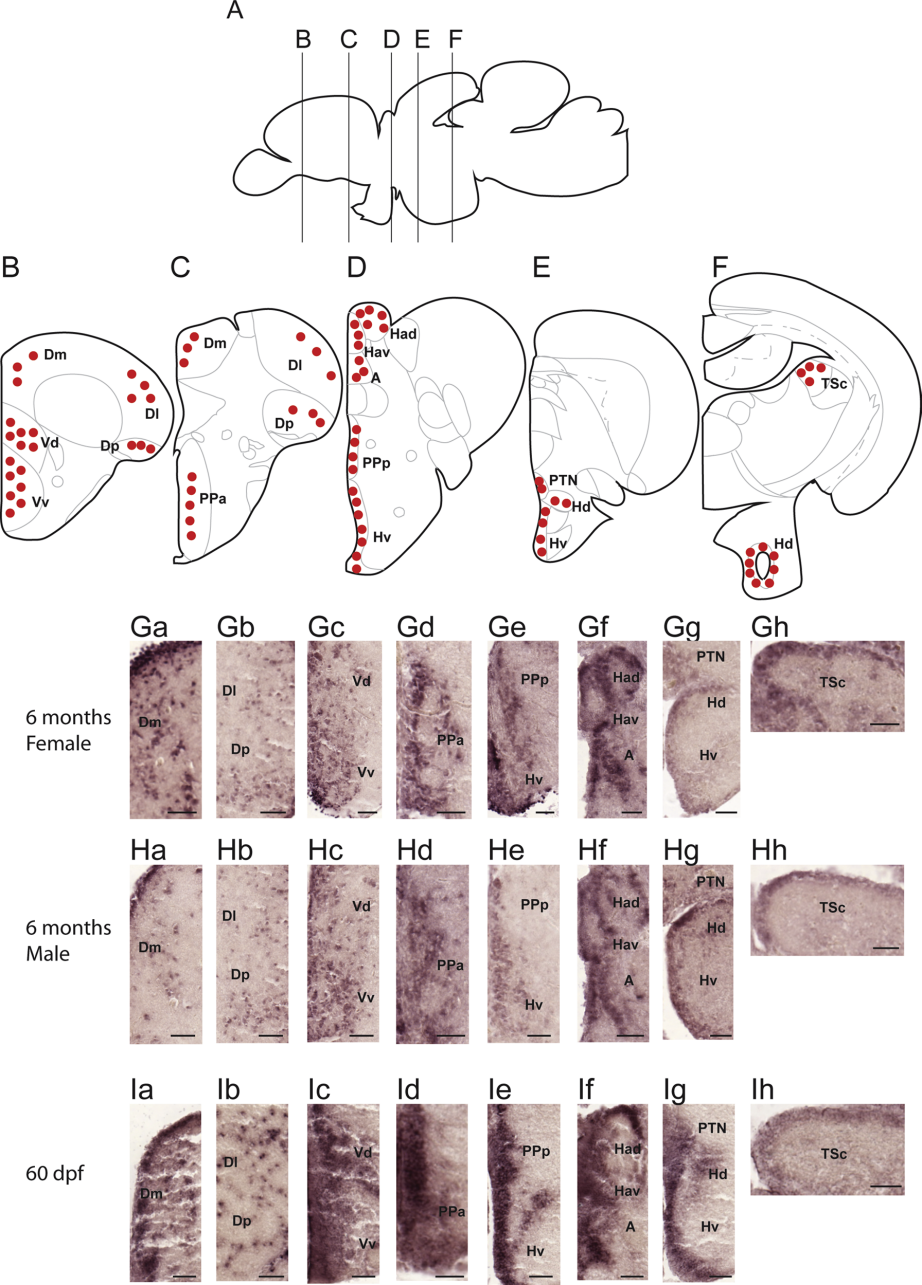
Localisation of *bpifcl* mRNA expression in zebrafish brain. (**A**) Schematic sagittal drawing of the zebrafish brain. (**B**–**F**) Lines in A indicate levels of coronal sections. Schematic coronal brain drawing of zebrafish showing the distribution of *bpifcl* (red dots) mRNA-containing cells in the brain. (**Ga**–**Gh**) Photomicrographs of *bpifcl*-expressing cells in 6 months old female zebrafish brain. (**Ha**–**Hh**) Photomicrographs of *bpifcl*-expressing cells in 6 months old male zebrafish brain. (**Ia**–**Ih**) Photomicrographs of *bpifcl*-expressing cells in 60 days post-fertilisation (dpf) fish brain. For abbreviations, see Supplementary Abbreviation. Scale bar: 50 μm.

Distributions of *bpifcl* mRNA in the 6 months old female zebrafish, 6 months old male fish and 60 days post-fertilization (dpf) fish were identified using digoxigenin (DIG)- *in situ* hybridisation (ISH). The general distribution pattern of *bpifcl* mRNA is illustrated in Fig. 1. Strong signals were detected in select regions of the brain: the lateral, medial and posterior zone of the dorsal telencephalic area (Fig. 1B,C, Ga, Gb, Ha, Hb, Ia and Ib), dorsal and ventral nucleus of the ventral telencephalic area (Fig. 1B, Gc, Hc and Ic), anterior and posterior parvocellular preoptic nucleus (Fig. 1C,D, Gd, Ge, Hd, He, Id and Ie), dorsal and ventral habenular nucleus (Fig. 1D, Gf, Hf and If), anterior thalamic nucleus (Fig. 1D, Gf, Hf and If), dorsal (Hd) and ventral zone of periventricular hypothalamus (Fig. 1D,E, Ge, Gg, He, Hg, Ie and Ig), posterior tuberal nucleus (Fig. 1E, Gg, Hg and Ig) and central nucleus of torus semicircularis (Fig. 1F, Gh, Hh and Ih). There were no distribution differences among 6 months old female, 6 months old male and 60 dpf fish.

### Association between *bpifcl* mRNA expression and *kiss2* mRNA expression

**Figure 2:**
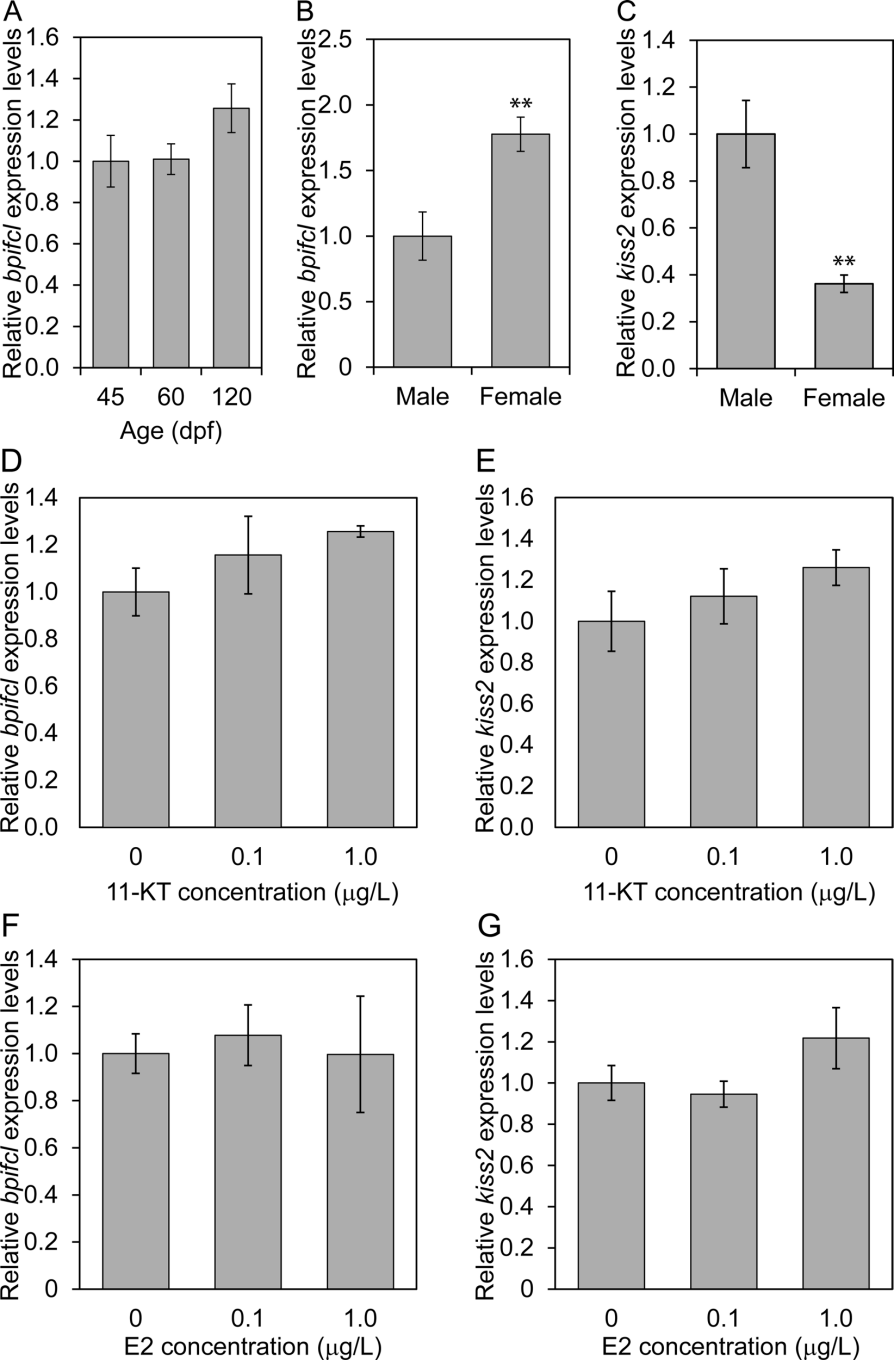
Association between *bpifcl* mRNA and *kiss2* mRNA expression. *bpifcl* mRNA expression in zebrafish brain during development (**A**) and between 6 months old males and females (**B**). *kiss2* mRNA expression between 6 months old males and females (**C**). Effect of 11-KT exposure on *bpifcl* (**D**) and *kiss2* (**E**) mRNA expression in 6 months old male zebrafish brain. Effect of E2 exposure on *bpifcl* (**F**) and *kiss2* (**G**) mRNA expression in 6 months old male zebrafish brain. Expression levels of 45 days post-fertilisation (dpf) (**A**), males (**B**,**C**) and 0 μg/L exposure (**D**–**G**) were defined as 1.0. All data are presented as the mean ± standard error of the mean and were analysed by a Student’s *t*-test for sex differences and one-way analysis of variance and Tukey’s post-hoc test for multiple comparisons for the others. **p < 0.01.

There was no significant difference in *bpifcl* mRNA expression between 45, 60 and 120 dpf zebrafish brains (Fig. 2A and Supplementary Table 2A). Meanwhile, *bpifcl* mRNA expression was significantly high in the 6 months old females compared with the 6 months old males (Fig. 2B and Supplementary Table 2B). On the other hand, *kiss2* mRNA was significantly low in the 6 months females (Fig. 2C and Supplementary Table 2C).

### Effect of 11-ketotestosterone (11-KT) and β-estradiol (E2) on *bpifcl* and *kiss2* mRNA expression

After 48 h exposure with E2 and 11-KT to the 6 months old males, *bpifcl* and *kiss2* mRNA expressions were not significantly different, regardless of concentration (Fig. 2D–G and Supplementary Table 2D–G). *gnrh3* mRNA expression was significantly different at 0.1 and 1.0 μg/L of E2 (Supplementary Fig. 2B and Supplementary Table 2O).

**Figure 3:**
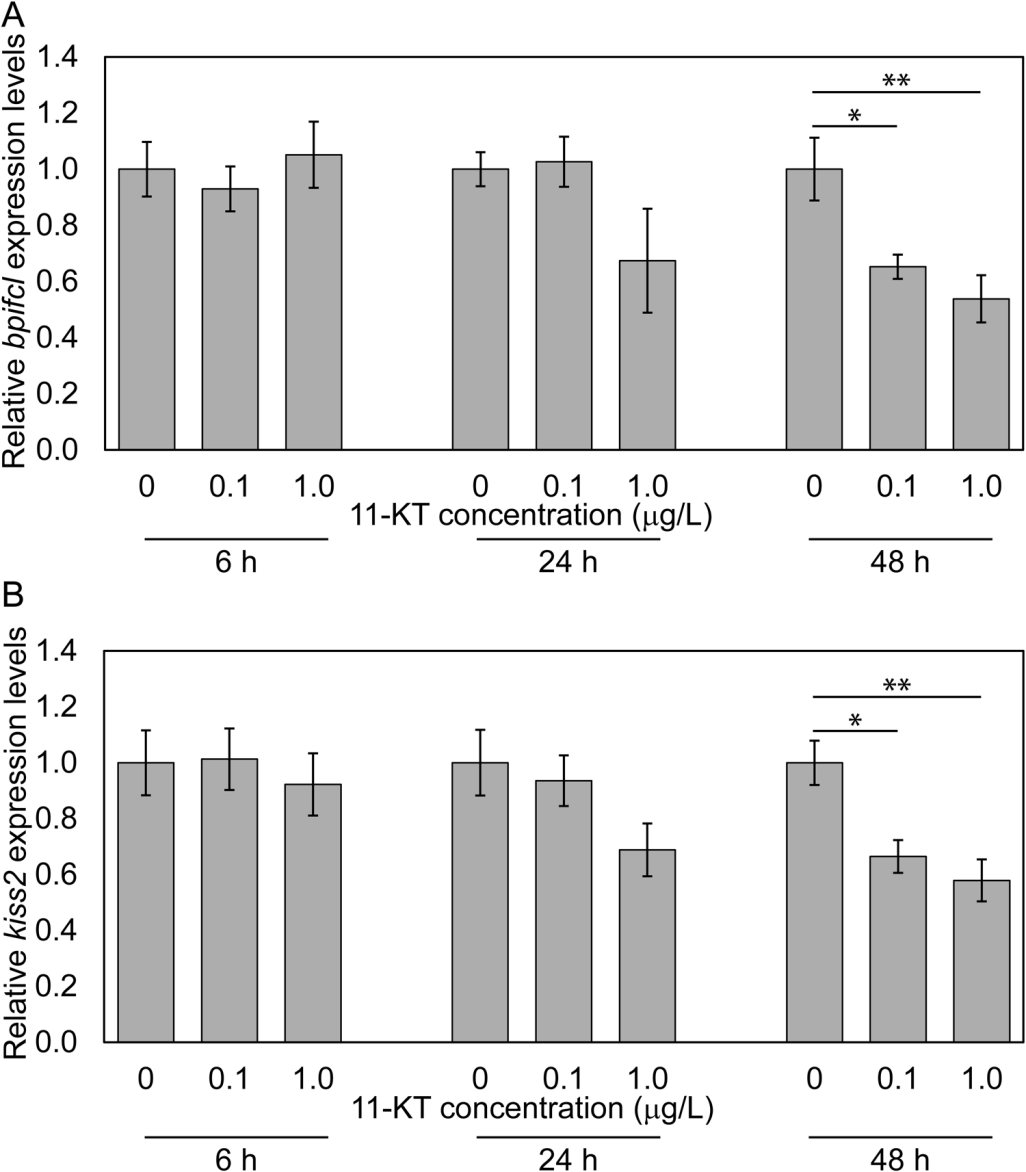
Effect of 11-KT on 6 months old female zebrafish. Effect of 11-KT on *bpifcl* (**A**) and *kiss2* (**B**) mRNA expression in 6 months old female zebrafish brains. Expression levels of 0 μg/L exposure at each time point (**A**,**B**) were defined as 1.0. All data are presented as the mean ± standard error of the mean and were analysed by a one-way analysis of variance and Tukey’s post-hoc test for multiple comparisons. *p < 0.05, **p < 0.01.

After 48 h exposure with 11-KT to the 6 months old females, *bpifcl* and *kiss2* mRNA expression were significantly lower at 0.1 and 1.0 μg/L, respectively (Fig. 3A,B, Supplementary Table 2H and I), however, *gnrh3* mRNA expression was not significantly different (Supplementary Fig. 2A and Supplementary Table 2N). *bpifcl* and *kiss2* mRNA expressions were not significantly different after 6 h and 24 h exposure to 11-KT, regardless of concentration (Fig. 3A,B, Supplementary Table 2H and I).

### Expression of *bpifcl* in KISS2 neurons and effect of knocked-down *bpifcl* mRNA expression

**Figure 4:**
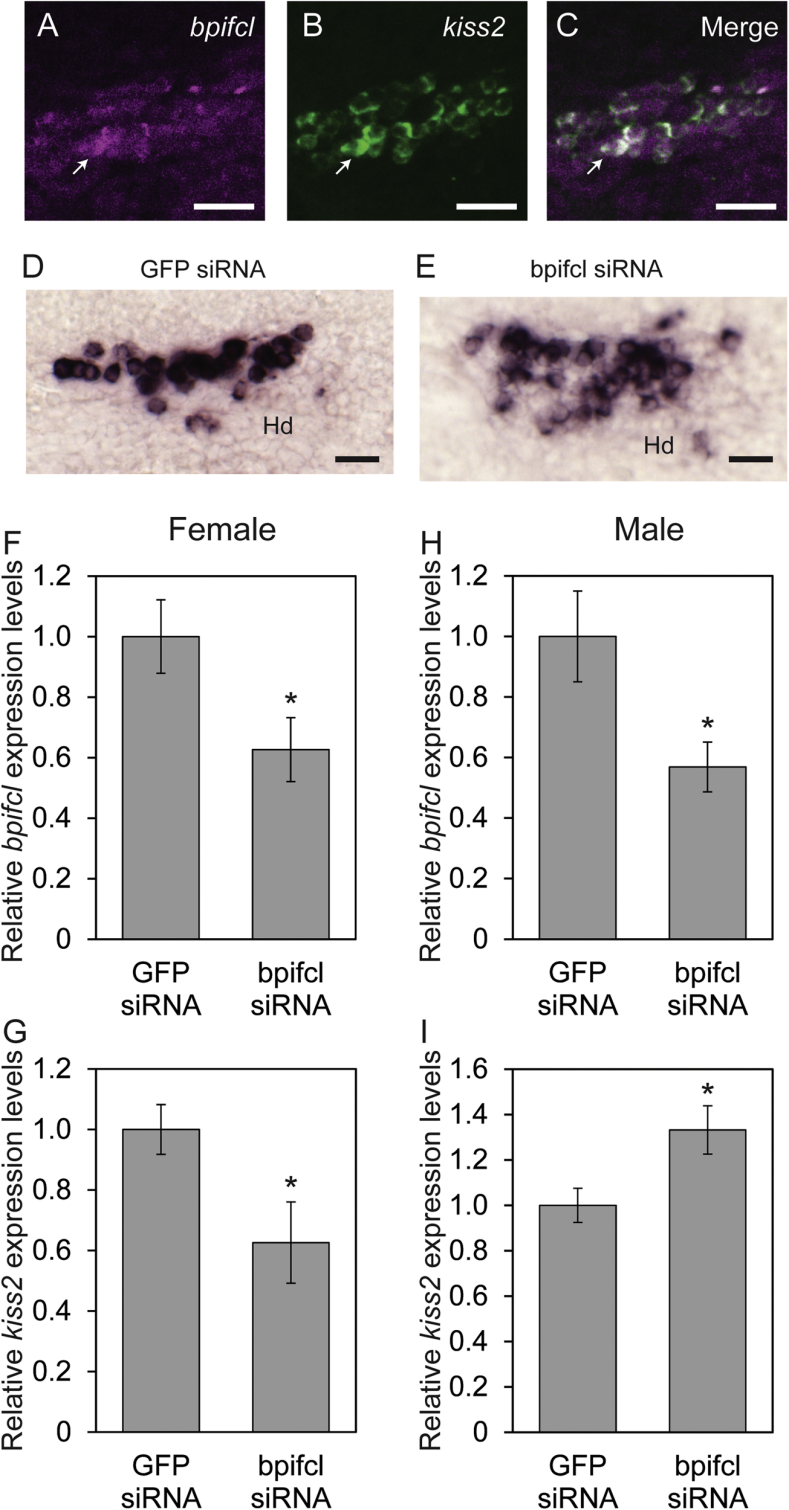
Photomicrographs of double-label fluorescence ISH of *bpifcl* and *kiss2* mRNA, and effect of knocked-down *bpifcl* mRNA expression. Anti-sense riboprobe-labelled *bpifcl* (**A**) and *kiss2* (**B**) mRNA was co-expressed in the dorsal zone of the periventricular hypothalamus (Hd) (**C**). Scale bars: 20 μm. Localisation of *kiss2* mRNA in the Hd of GFP siRNA injected female (**D**) and *bpifcl* siRNA injected female (**E**). Scale bars: 20 μm. *bpifcl* (**F**) and *kiss2* (**G**) mRNA expression in *bpifcl* siRNA-injected female zebrafish, and *bpifcl* (**H**) and *kiss2* (**I**) mRNA expression in *bpifcl* siRNA-injected males. Expression levels of GFP siRNA injected fish were defined as 1.0. All data are presented as the mean ± standard error of the mean and analysed by a Student’s *t*-test. *p < 0.05.

*bpifcl* mRNA expression in KISS2 neurons of the Hd was examined using double-label fluorescence ISH. All KISS2 neurons expressed *bpifcl* in the Hd (Fig. 4A–C). Furthermore, the effect of knocked-down *bpifcl* expression on *kiss2* mRNA expression was also examined. All siRNA administrated fish survived. No localisation difference of KISS2 neurons in 6 months old female fish was exhibited by *bpifcl* specific siRNA administration (Fig. 4D and E). *bpifcl* mRNA expression was confirmed as significantly decreased in *bpifcl* small interfering RNA (siRNA)-injected female (Fig. 4F and Supplementary Table 2J) and male (Fig. 4H and Supplementary Table 2L) zebrafish compared to green fluorescent protein (GFP) siRNA-injected fish. Since suppression of *bpifcl* mRNA expression was confirmed, *kiss2* mRNA expression was also examined. *kiss2* mRNA expression was also significantly lower in *bpifcl* siRNA-injected female zebrafish (Fig. 4G and Supplementary Table 2K), but was significantly higher in *bpifcl* siRNA-injected males (Fig. 4I and Supplementary Table 2M) compared to GFP siRNA-injected fish.

## Discussion

In the current study, an isoform of *bpifcl* expressed in the brain of zebrafish was identified. Functional domain analysis using SMART showed the presence of N- and C-terminal domain of the BPIF family. Furthermore, the three-dimensional structure of the BPIFCL protein predicted by SWISS-MODEL (http://swissmodel.expasy.org) using human BPI as a template revealed a boomerang-shaped molecule with two twisted, anti-parallel β-sheets and two α-helices at both the N- and C-terminals, as well as the presence of two distinct barrel-shaped domains (data not shown). These results indicate that the identified *bpifcl* isoform belongs to the BPIF family of genes expressed in the brain.

*bpifcl* mRNA expression in mature female zebrafish brains was significantly higher than in mature males. On the other hand, *kiss2* mRNA expression in mature females was significantly lower than in mature males. This suggests that sex steroids affect both of *bpifcl* and *kiss2* mRNA expressions. During development, particularly around puberty (45 and 60 dpf), and matured fish (120 dpf), no significant difference of *bpifcl* mRNA expression was revealed. *kiss2* mRNA expression is also remained at high levels during this period^22^, and localisation of *bpifcl* mRNA in the 60 dpf fish brain including the Hd where KISS2 neurons are present^22^ was not significantly different from mature fish. *bpifcl* mRNA expression could be associated with *kiss2* mRNA expression.

Since sex steroids suggest to affect *bpifcl* and *kiss2* mRNA expressions, fish were exposed to E2 and 11-KT, and *bpifcl* and *kiss2* expressions were examined. Sex steroids affect sexual behaviour and spawning when male zebrafish are exposed to E2 and female fish are exposed to 11-KT^23^, therefore, *bpifcl* mRNA expression was expected to be altered by these exposure. Male fish exposed to E2 revealed no significant difference in *kiss2* or *bpifcl* expression. On the other hand, females exposed to 11-KT revealed significantly lower levels of *kiss2* and *bpifcl* mRNA expression after 48 h exposure, however, 11-KT exposure to male did not affect *bpifcl* and *kiss2* mRNA expression. Since both of *bpifcl* and *kiss2* mRNA expressions were decreased by 11-KT exposure, we administered *bpifcl* siRNA to evaluate involvement of *bpifcl* in the regulation of *kiss2* mRNA expression. In both male and female zebrafish brains, *bpifcl* mRNA expression was successfully suppressed by *bpifcl* siRNA compared to GFP siRNA-injected fish. Generally, siRNA transfection *in vitro* leads to more than 70% suppression of the target gene^24, 25^, whereas *in vivo* administration commonly leads to 30–50% suppression^26^. Suppression of about 30% in the current study was comparable to *in vivo* siRNA administration reported in other species. *bpifcl* siRNA administration herein decreased *kiss2* mRNA expression in female fish but not the number of KISS2 neurons. Androgen receptors are widely expressed in the Hd of zebrafish^27^, comparable to *bpifcl* expression identified in this study; this strongly suggests that testosterone could regulate Bpifcl modulation of *kiss2* mRNA expression in the Hd of females. The BPIF family is known as a secreted protein^3, 5–10^, however, several genes such as CETP^28^ and PLTP^29^ are also expressed in cytoplasm and nucleus. Especially, PLTP is suggested to be a modulator of signal transduction of the activation of the phosphatidylinositol-3 kinase/Akt pathway^29^. In this study, localisation of Bpifcl in the cell was not identified, however, the phylogenetic tree analysis indicated that Bpifcl is closer to Pltp than Cetp suggesting that Bpifcl could also play a modulatory role in signal transduction pathways. A signal transduction pathway of androgen to *kiss2* gene has not been identified, however, Bpifcl could be involved in modulation of the pathway. In this study, *kiss2* mRNA expression in male fish exposed to 11-KT was not affected. Male zebrafish exposed to 11-KT do not change sexual behaviour^23^. This suggests that male fish which high levels of testosterone are endogenously expressed^30^ are not affected by external exposure, therefore, *kiss2* mRNA expression might be unaffected by 11-KT exposure.

*kiss2* neurons are present in the Hd, Hv and posterior tuberal nucleus (nPT)^22^. *kiss2* mRNA was not detected in the Hv in the current study. Since Servili and coworkers^31^ showed no *kiss2* neurons in the Hv, localisation of *kiss2* neurons in the Hv is still debatable. In the nPT, *bpifcl* mRNA was not expressed. Therefore, *kiss2* and *bpifcl* mRNA could be co-localised in only the Hd. Interestingly, *kiss2* mRNA expression was significantly increased by *bpifcl* siRNA administration in males. Meanwhile, neither E2 nor 11-KT affected to *kiss2* mRNA expression in male fish. These suggest that Bpifcl is involved in several regulatory mechanisms of *kiss2* mRNA expression and differently involved in the regulation of *kiss2* mRNA expression between male and female. We identified Bpifcl as a regulator of *kiss2* mRNA expression in female. This regulatory mechanism could be involved in testosterone-related physiologies other than reproduction. Since androgens mediate inflammation in the brain^32^, this regulatory mechanism could respond to inflammation in the brain.

In vertebrates, the main reproductive regulatory system is the production and secretion of gonadotropin-releasing hormone (GnRH) from GnRH3 neurons in the preoptic area. GnRH regulates gonadal maturation by stimulating the synthesis and secretion of gonadotropins^33^. Kisspeptin, a product of *kiss* expression, is a critical regulator of GnRH secretion^15^. In the current study, exposure of female zebrafish to 11-KT did not affect *gnrh3* mRNA expression. In female mummichogs, exposure to 5α-dihydrotestosterone for 21 days was shown to affect fecundity, particularly low egg production^34^. Trenbolone acetate, a synthetic androgen, was also shown to decrease ovarian follicles in female Japanese medaka after 14 days of exposure^35^. Here, female zebrafish were exposed to 11-KT for 48 h to investigate any direct correlation(s) with *bpifcl*. Either this exposure time is too short to affect *gnrh3* mRNA expression or kisspeptin might not regulate *gnrh3* in zebrafish. In fact, a previous study showed that knocking-out *kiss2* in zebrafish did not affect their reproductive capability^36^. Here we found *gnrh3* mRNA expression was significantly increased in male fish exposed to E2. *gnrh3* mRNA expression is affected by E2 in teleosts^37^, and oestrogen receptors are expressed in the preoptic area, where GnRH neurons are present in fish^38^. Therefore, current E2 results suggest a direct effect on *gnrh3* mRNA expression.

An orthologous gene to zebrafish *bpifcl* has not been identified in other species. However, BPIF genes have been estimated in other species, such as humans (NCBI gene ID: 254240), mouse (270757), rat (299685), bird (107204354), turtle (102945958) and snake (106539050). Furthermore, as we have identified that several BPIF family genes are expressed in the brain and affected by ageing and chronic selective serotonin reuptake inhibitor treatment^25^. Therefore, an orthologous gene to zebrafish *bpifcl* could be encoded in other species. Furthermore, modulation of kisspeptin by Bpifcl might be a conserved function in vertebrates since kisspeptin is conserved in vertebrates.

In the current study, we showed involvement of a BPIF family gene regulates gene expression in the zebrafish brain. Although many BPIF family genes are estimated in mammals^5^, most of the BPIF family genes have unknown function. Some BPIF family genes could be involved in the regulation of gene expression, like *bpifcl*. The current results suggest that testosterone regulates Bpifcl modulation of *kiss2* expression in the Hd of zebrafish, particularly in females. Considering we found that *bpifcl* expression was widely distributed in the brain, it is reasonable to posit that Bpifcl plays a role in regulating other neurons, particularly those regulated by testosterone, such as KISS2 neurons.

## Methods

### Animals

Zebrafish (*Danio rerio*) were maintained in fresh water at 27.0 ± 1.0 °C under a controlled natural photo regimen (14-h light/10-h dark cycle). Fish were fed an Adult Zebrafish Diet (Zeigler Bros., PA, USA) twice daily. To obtain developing embryos, one pair of fish was placed in a tank with glass marbles overnight to allow mating. Fertilised eggs were then syphoned from the tank and maintained at 27.0 ± 1.0 °C. After hatching, larvae were fed paramecium twice daily for the first 3 weeks and brine shrimps (Zebrafish Management Ltd., Hampshire, UK) twice daily for the following 4 weeks. This study was approved by the Animal Ethics Committee of Monash University (Melbourne, Australia; Approval no. MARP/2012/147). All experimental procedures were performed in accordance with guidelines of the Animal Ethics Committee of Monash University.

### Cloning of full-length *bpifcl* cDNA

Zebrafish were anaesthetised by immersing them in a 0.01% solution of benzocaine (Sigma, MO, USA) and killed by decapitation in order to dissect the fresh brains. Total RNA was isolated from whole brain with TRIzol reagent (Invitrogen, CA, USA) and converted to cDNA with SuperScript III (Invitrogen) and oligo d(T) primers. A partial coding sequence region was amplified by polymerase chain reaction (PCR). The primers were designed based on the genomic sequence of a predicted zebrafish *bpifcl* gene (Accession no. ENSDARG00000099980); primer sequences were as follows: forward, 5′-ATGCAGAGGCTGATGTTCCTCCTG-3′; reverse, 5′-TTATGGAGCATTCAGCCCATCAG-3′. The PCR reaction was performed in a 20 μL reaction mixture containing 1X HotStarTaq Master Mix (Qiagen, Hilden, Germany), 0.5 μM of each primer and 1 μL of cDNA. The amplified DNA was subjected to direct sequencing.

The full-length *bpifcl* mRNA sequence was identified by 3′- and 5′-rapid amplification of cDNA ends (RACE). Gene-specific primers were designed based on the identified partial mRNA sequence. For 3′-RACE, 1 μg of total RNA was converted to cDNA with SuperScript III and 50 pmol of oligo d(T)-containing adapter primer in a 20 μL reaction volume. The converted cDNA was subjected to PCR in a 10 μL reaction volume containing 1X HotStarTaq Master Mix, 1 pmol of abridged universal amplification primer (Invitrogen) and 10 pmol of a gene-specific primer (5′-TGAGGTCCGGCTTTCTCTTGCCCAC-3′).

For 5′-RACE, 1 μg of the total RNA was converted to cDNA in a 20 μL reaction volume with SuperScript III and 2 pmol of a gene-specific primer (5′-ACAGTCCTGTTCCCTCCACAAACAC-3′). The polycytosine-tailed cDNA with terminal transferase (Roche Diagnostics, Mannheim, Germany) was subjected to PCR in a 20 μL reaction volume containing 1X HotStarTaq Master Mix, 1 pmol of 5′-RACE abridged anchor primer (Invitrogen) and 10 pmol of a gene-specific primer (5′-CGGCTCAGCTGTTTCGCACTGCACG-3′). Furthermore, 1 μL of the PCR reaction mixture was subjected to nested PCR in a 20 μL reaction mixture containing 1X HotStarTaq Master Mix, 1 pmol of abridged universal amplification primer and 10 pmol of a gene specific primer (5′-TATCCAGACAGGCATCATCTGAGAC-3′). The complete nucleotide sequence of *bpifcl* mRNA was confirmed by sequence analysis of each PCR product. The first ATG codon with a Kozak translation initiation sequence was considered the initial codon^39, 40^. Functional domain regions were further analysed by SMART^41, 42^.

### Localisation of *bpifcl* mRNA in adult zebrafish brain using ISH

6 months old male zebrafish, 6 months old female fish and 60 dpf fish were anaesthetized by immersion in 0.01% benzocaine solution and killed by decapitation in order to dissect the fresh brains. The brains were fixed in buffered 4% paraformaldehyde for 6 h, cryoprotected in 20% sucrose and then embedded in Tissue Tek OCT compound (Sakura Finetechnical, Tokyo, Japan). Coronal sections (12 μm thick) were cut on a cryostat and thaw-mounted onto 3-aminopropylsilane-coated glass slides. The RNA probes were synthesised by *in vitro* transcription from a pGEM-T Easy vector (Promega, Madison, WI, USA), containing sequences amplified with a forward 5′-CGAACCGTGAACATACCCGT-3′ and reverse 5′-TCTGTCGAGCGCCTGTAGA-3′ primer. Sense and anti-sense DIG-labelled riboprobes were synthesised using MAXIscript (Ambion, Austin, TX, USA) and DIG RNA Labeling Mix (Roche Diagnostics). DIG-ISH was performed as described previously^22, 43^ with minor modifications. Brain regions were followed according to the neuroanatomy of the zebrafish brain^44^.

### Double-label fluorescence ISH of *bpifcl* and *kiss2* mRNA

Riboprobes for *bpifcl* and *kiss2* ^22^ mRNA were labelled with biotin and DIG, respectively, using MAXIscript with either biotin or DIG RNA Labeling Mix (Roche Diagnostics). The biotin-labelled *bpifcl* probe was detected using horseradish peroxidase-streptavidin and AlexaFluor 594 Tyramide (Invitrogen), whereas the DIG-labelled *kiss2* probe was detected using a Tyramide Signal Amplification Plus kit (PerkinElmer/NEN Life Science Products, MA, USA). The biotin-labelled *bpifcl* and DIG-labelled *kiss2* riboprobes were mixed in a cocktail for the hybridisation. After hybridisation, a peroxidase-conjugated anti-DIG antibody (Roche Diagnostics) diluted 1:500 in a buffer containing 0.1 M Tris (pH 7.4), 0.15 M NaCl and Tween buffer (0.05% Triton X-100) with 1% normal goat serum was applied to each slide for 2 h at room temperature, followed by incubation with Tyramide Signal Amplification Working Solution for 1 min for colour development of *kiss2* probes. After blocking with 3% H_2_O_2_ in a 0.1 M Tris-HCl (pH 7.4) and 0.15 M NaCl buffer for 30 min at room temperature, a horseradish peroxidase-conjugated streptavidin antibody diluted 1:100 in the 0.1 M Tris-HCl (pH 7.4) and 0.15 M NaCl buffer buffer with 2% bovine serum albumin was applied to each slide overnight at 4 °C. The colour development reaction for *bpifcl* probes was initiated by adding a 1:100 dilution of reconstituted AlexaFluor 594 Tyramide to the Amplification Buffer (Invitrogen) with 0.0015% H_2_O_2_ for 15 min. Separate images were captured with the appropriate excitation wavelengths, and computer software (NIS Elements; Nikon, Tokyo, Japan) was used to superimpose the two images.

### *bpifcl* mRNA expression analysis during development in male and female zebrafish

Zebrafish at 45 dpf (*n* = 13), 60 dpf (*n* = 12), 120 dpf (female, *n* = 8; male, *n* = 6) and 6 months old males (*n* = 10) and females (*n* = 10) were anaesthetised by immersing them in 0.01% benzocaine solution, followed by decapitation and fresh brain dissection. Standard lengths of the fish were not measured. Total RNA was isolated from whole brains with TRIzol reagent and converted to cDNA with a High Capacity cDNA Reverse Transcription Kit (Applied Biosystems). Levels of β-actin, *bpifcl* and *kiss2* mRNA were examined by real-time PCR. Real-time PCR was performed in 10 μL reaction mixtures containing 1X SensiFAST SYBR Master Mix (BioLine Reagent, London, UK), 0.2 μM of each forward and reverse primer and 1 μL of cDNA. The primers used are shown in the Supplementary Table 1. Real-time PCR was performed using a 7500 Fast PCR system (Applied Biosystems) with conditions of 95 °C for 10 min, 40 cycles of 95 °C for 15 s and 60 °C for 1 min, followed by a dissociation step. The levels of each mRNA type were normalised to *β-actin* mRNA using the ΔΔCt method.

### Effect of E2 and 11-KT on *bpifcl* and *kiss2* expression

6 months old male zebrafish were exposed to either 0.01% dimethyl sulfoxide (DMSO, Sigma) and 0.1 or 1.0 μg/L E2 in 0.01% DMSO for 48 h, and 6 months old female fish were exposed to 0.01% DMSO and 0.1 or 1.0 μg/L 11-KT in 0.01% DMSO. Fish were randomly selected and divided into three groups (*n* = 10 per group). The treatment was performed in tanks [280 mm (L) × 210 mm (W) × 180 mm (H)] containing 2 L of the above solution. During the experiment, the solution was changed after 24 h. The 0.1 μg/L dose of E2 was selected because it has been shown to affect the reproductive system of male zebrafish^45, 46^. The corresponding 11-KT concentrations were selected to match and compare with E2 doses. During exposure, fish were fed an Adult Zebrafish Diet (Zeigler Bros.,) twice daily. After exposure, *β-actin*, *bpifcl*, *kiss2* and *gnrh3* expression was examined as described above. The primers used are shown in the Supplementary Table 1.

### Effect of knocked-down *bpifcl* expression on *kiss2* mRNA expression

Custom-designed *in vivo* siRNA (Ambion), *bpifcl*-specific siRNA (sense, 5′-GGAACGAACCGUGAACAUAtt-3′; anti-sense, 5′-UAUGUUCACGGUUCGUUCCtg-3′) or GFP-specific siRNA (sense, 5′-GCAUCAAGGUGAACUUCAAtt 3′; anti-sense, 5′-UUGAAGUUCACCUUGAUGCcg-3′) was intracranially administered to the 6 months old zebrafish. *bpifcl* or GFP siRNA (60 pmol/µL) was mixed with the same volume of 10% glucose and 0.06 volume of Turbofect (Thermo Scientific, MA, USA) and incubated for 20 min at room temperature. Anaesthetised fish with 0.01% benzocaine solution (*n* = 10 per group) were placed on a sponge soaked with water, the skulls punctured with a 25 G × 1-in needle (Terumo, Tokyo, Japan) in the midline at the telencephalon-diencephalon border^47^ and 1.0 µL of the mixture intracranially injected. The fish were placed to recover in static water and fed an Adult Zebrafish Diet (Zeigler Bros.,) twice daily. After 48 h maintenance, *β-actin*, *bpifcl* and *kiss2* expression were examined by real-time PCR as described above. GFP siRNA-injected fish were used as negative controls.

### Statistical analysis

Statistical analysis of gene expression during development, by gender and for 11-KT and E2 exposure was performed using a one-way analysis of variance and Tukey’s post-hoc test for multiple comparisons using SPSS 20 software. A Student’s *t*-test was applied for sex differences and *bpifcl* knock-down experiments. *p* < 0.05 was considered significant.

## Electronic supplementary material

Supplementary information
