# Supplementary material for: Bpifcl modulates kiss2 expression under the influence of 11-ketotestosterone in female zebrafish
Source: Sci Rep. 2017 Aug 11;7:7926. doi: 10.1038/s41598-017-08248-8 (PMC5554142; doi:10.1038/s41598-017-08248-8)
Supplement: Supplementary file 1 — Supplementary information [file 41598_2017_8248_MOESM1_ESM.pdf]

Bpifcl modulates *kiss2* expression under the influence of 11-ketotestosterone in female zebrafish

Shogo Moriya*, Nabila Tahsin, Ishwar S. Parhar

Supplementary Abbreviation

11KT: 11-ketotestosterone

A: anterior thalamic nucleus (A)

Aβ: amyloid β (Aβ)

BPI: bactericidal/permeability-increasing protein

BPIF: bactericidal/permeability-increasing fold containing

Bpifcl; BPI fold containing family C, like

CETP: cholesterol ester transfer protein

Dl: lateral zone of dorsal telencephalic area

Dm: medial zone of dorsal telencephalic area

Dp: posterior zone of dorsal telencephalic area

E2: β-estradiol

Had: dorsal habenular nucleus

Hav: ventral habenular nucleus

Hd: dorsal zone of periventricular hypothalamus

HDL: high-density lipoproteins

Hv: ventral zone of periventricular hypothalamus

LBP: lipopolysaccharide-binding protein

LPS: lipopolysaccharide

nPT: posterior tuberal nucleus

PLTP: phospholipid transfer protein

PLUNC: palate, lung and nasal epithelium clone

POA: preoptic area

PPa: parvocellular preoptic nucleus, anterior part

PPp: parvocellular preoptic nucleus, posterior part

PTN: posterior tuberal nucleus

RACE: 3’- and 5’ - rapid amplification of cDNA end

SMART: simple modular architecture research tool

SSRI: selective serotonin reuptake inhibitors

TSc: central nucleus of torus semicircularis

Vd: dorsal nucleus of ventral telencephalic area

Vv: ventral nucleus of ventral telencephalic area

Supplementary Table 1. List of primers used for real-time PCR

Gene name	Sequence (5’ to 3’)	Product size (bp)
bpifcl	CGTGAACATACCCGTGGATAAG<br>CCTCTAATCGGCAGCTCTGATC	89
kiss2	GCCTATGCCAGACCCCAAA<br>TTTACTGCGTGCTAGTCGATGTTT	154
gnrh3	ATGGAGGCAACATTCAGGATGT<br>CCTTTCAGAGGCAAACCTTCA	131
β-actin	CCTGACAGAGCGTGGCTACTC<br>TCTCTTTGATGTCACGCACGAT	100

Supplementary Table 2. Descriptive statistics of each graph.

A. Descriptive statistics of Figure 2A.

	45 dpf	60 dpf	120 dpf
Mean	1.00	1.01	1.25
Standard deviation	0.48	0.29	0.46
95% Confidence interval	0.29	0.15	0.23

B. Descriptive statistics of Figure 2B.

	Male	Female
Mean	1.00	1.78
Standard deviation	0.71	0.49
95% Confidence interval	0.37	0.26

C. Descriptive statistics of Figure 2C.

	Male	Female
Mean	1.00	0.36
Standard deviation	0.45	0.12
95% Confidence interval	0.28	0.07

D. Descriptive statistics of Figure 2D.

	0 μg/L	0.1 μg/L	1.0 μg/L
Mean	1.00	1.16	1.26
Standard deviation	0.32	0.52	0.08
95% Confidence interval	0.26	0.32	0.08

E. Descriptive statistics of Figure 2E.

	0 μg/L	0.1 μg/L	1.0 μg/L
Mean	1.00	1.12	1.26
Standard deviation	0.46	0.42	0.27
95% Confidence interval	0.28	0.26	0.17

F. Descriptive statistics of Figure 2F.

	0 μg/L	0.1 μg/L	1.0 μg/L
Mean	1.00	1.08	1.00
Standard deviation	0.25	0.36	0.70
95% Confidence interval	0.16	0.22	0.43

G. Descriptive statistics of Figure 2G.

	0 μg/L	0.1 μg/L	1.0 μg/L
Mean	1.00	0.95	1.21
Standard deviation	0.27	0.20	0.47
95% Confidence interval	0.17	0.12	0.29

H. Descriptive statistics of Figure 3A.

	6 h			24 h			48 h		
	0 μg/L	0.1 μg/L	1.0 μg/L	0 μg/L	0.1 μg/L	1.0 μg/L	0 μg/L	0.1 μg/L	1.0 μg/L
Mean	1.00	0.93	1.05	1.00	1.03	0.67	1.00	0.65	0.54
Standard deviation	0.31	0.25	0.37	0.19	0.28	0.59	0.35	0.14	0.27
95% Confidence interval	0.19	0.16	0.23	0.12	0.18	0.36	0.23	0.09	0.17

I. Descriptive statistics of Figure 3B.

	6 h			24 h			48 h		
	0 μg/L	0.1 μg/L	1.0 μg/L	0 μg/L	0.1 μg/L	1.0 μg/L	0 μg/L	0.1 μg/L	1.0 μg/L
Mean	1.00	1.01	0.92	1.00	0.94	0.69	1.00	0.66	0.58
Standard deviation	0.36	0.35	0.35	0.37	0.29	0.30	0.25	0.19	0.24
95% Confidence interval	0.23	0.22	0.22	0.23	0.18	0.18	0.16	0.12	0.15

J. Descriptive statistics of Figure 4F.

	GFP siRNA	bpifcl siRNA
Mean	1.00	0.63
Standard deviation	0.30	0.32
95% Confidence interval	0.24	0.25

K. Descriptive statistics of Figure 4G.

	GFP siRNA	bpifcl siRNA
Mean	1.00	0.63
Standard deviation	0.23	0.33
95% Confidence interval	0.19	0.26

L. Descriptive statistics of Figure 4H.

	GFP siRNA	bpifcl siRNA
Mean	1.00	0.57
Standard deviation	0.45	0.25
95% Confidence interval	0.36	0.20

M. Descriptive statistics of Figure 4I.

	GFP siRNA	bpifcl siRNA
Mean	1.00	0.33
Standard deviation	0.23	0.32
95% Confidence interval	0.18	0.26

N. Descriptive statistics of Supplementary Figure 2A.

	0 μg/L	0.1 μg/L	1.0 μg/L
Mean	1.00	1.03	0.85
Standard deviation	0.33	0.33	0.29
95% Confidence interval	0.21	0.22	0.19

O. Descriptive statistics of Supplementary Figure 2B.

	0 μg/L	0.1 μg/L	1.0 μg/L
Mean	1.00	7.70	6.05
Standard deviation	0.30	2.05	2.31
95% Confidence interval	0.19	1.27	1.43

A

TCAGTCGGACGCTCAGACAGAAGCAGATGAAGTTCATTAGTTCATTACTGCTGTAATTTG
CAGGATGCAGAGGCTGATTTTCCTCCTGATGTTGACGCAGTCATGTGCGGATAATCCAGC
M Q R L I F L L M L T Q S C A D N P A
ATTCAAAGCTCTTCTGTCAGAGAAGGCTCTCACAGACTTGTCTCAGATGATGCCTGTCTG
F K A L L S E K A L T D L S Q M M P V W
GATACAAAGCAAAATGAAGAGCACCGCAATCCCAGACATCCATGATCAAGTGGACATTGG
I Q S K M K S T A I P D I H D Q V D I G
CATCGGCTGGGTGAACTACGTCCTCTCACACATGCGTGTCGTGCAGTGCGAAACAGCTGA
I G W V N Y V L S H M R V V Q C E T A E
GCCGTCGCTGGTGTTTGTGGAGGGAACAGGACTGTATCTGGAGGTCCGTGAGCTCTCATT
P S L V F V E G T G L Y L E V R E L S L
GGCTGTTTCTGGCAGATGGAGAACAAAGTTCGGCATCATCACAGACAGCGGCTCGTTTGA
A V S G R W R T K F G I I T D S G S F D
TGTGGAGGTCTACAATATATACATCCGTGTGGTCCTGGGGGTCGGGGATAAAGACGGTCA
V E V Y N I Y I R V V L G V G D K D G H
CCTCTCCATCAGCAGCGAATCCTGCAGTAATGATGTAGGAAATGTCTACATTCAGTTTCA
L S I S S E S C S N D V G N V Y I Q F H
CGGAGGAACAAGCTTCTTCTATCAGCTGTTTGAAGATTATTTCAGTGGAAAAGCCTCAGA
G G T S F F Y Q L F E D Y F S G K A S D
CATGATACGCCAGAAGATCTGTCCAGCAATCCAACAAGCAGTCACTAACATGGAGACAAT
M I R Q K I C P A I Q Q A V T N M E T I
TCTGCAGGAACGAACCGTGAACATACCCGTGGATAAGTATGTTTATCTGAGTGCTCCCCT
L Q E R T V N I P V D K Y V Y L S A P L
AACCTCCGCCCCGGCCGTGACCGATCAGAGCTGCCGATTAGAGGTTAAGGCTGAGTTTTA
T S A P A V T D Q S C R L E V K A E F Y
CAGCAGGCGTTCTCCATCTGAACCTCCATTCTCCGCCCGTGCGTTTGACCTTCAGTATTC
S R R S P S E P P F S A R A F D L Q Y S
AGACAAGCACATGCTTACACTGGCTGCATCCCAGTTCACGGTCAACTCAGCAGCGTTTGC
D K H M L T L A A S Q F T V N S A A F A
GTATCTCAGATCTGGGGCTCTTCAAACCAACATCACAGACGACATGATACCAAAGGGTTC
Y L R S G A L Q T N I T D D M I P K G S
CCCGCTGCATCTGAACACCAGTCAGTTTGGGGTCTTCATCCCACAGCTGCGCACATTATA
P L H L N T S Q F G V F I P Q L R T L Y
CCCCGACATGAAGATGCAGGTGCTGTTATACGCCAGCGACATGCCGCTGTTCTCCTTCAC
P D M K M Q V L L Y A S D M P L F S F T
TTCAGGCCTCATGAACATTCATGTGAAGATGGCAGCAAAGTTCTCCGCTGTTAAAGCTGA
S G L M N I H V K M A A K F S A V K A D
CGACGCTCTGGTGCCTCTTTTCACACTGAATGTGGACAGCAGATTCAGCGGAATCGCTCA
D A L V P L F T L N V D S R F S G I A Q
GATTAGCAATCAAAAACTGACCGGAGCTTTCAAAGTGAACAATATAACACTGACTGTGGG
I S N Q K L T G A F K V N N I T L T V G
ATCATCTGAGATTGGAGATTTCAAGACTGACACAATTAGGCAAGTGTTAGTGATCGCCGT
S S E I G D F K T D T I R Q V L V I A V
CAACACCATTATCCTGCCAAAGCTAAACGCTCGTCTGAGGTCCGGCTTTCTCTTGCCCAC
N T I I L P K L N A R L R S G F L L P T
GCTGCAGGGATTTAGCTTGAGTAACTCTCAACTGCTCATCAAAAACGGCTTTGTGGTCAT
L Q G F S L S N S Q L L I K N G F V V I
TTTTACCGACATCAGACTCCCTGATGGGCTGAATGCTCCATAACGGCTCACATGTCAACA
F T D I R L P D G L N A P
CAATCTACAGGCGCTCGACAGAACTCTGCTGTTCATTATATAGCCAACTGCTTTTGTGTT
GTCATGTTTTGTGTGAACAAACTGCTTATACGTTTCCCAGTGCTGGGTTGCTGCTGGAAG
GGCATCCACTGCCTAAAAAATACGCTGGAATAGTTGGCGGATGGTTCCGCTGTGGTGACC
CCTGATGAACCAAGGGAAAATGAATGAACTGCTGTCATTGGCTGTGTGTTGTTGTCTCTT
GTTTTGTGAGCACACGGCTTTGTTTTGTTTCACTTTCTGCCATGCACTTTCATCAGTCTC
CCCCGTCTCATCTCTTACTGTTACCTGACACATCTCCAAATTACCTAGGGACGACTGGCT
GTAATTCTCCATTCCAGTGACTAGGGCTGGGTATCATATGAATGTTATCAATTCTGATTA
AAGTTACCAATTTTTTCGGTTAAAAAAAAAAAAAAAAAAAAAAAAAAAAAAAAA

B

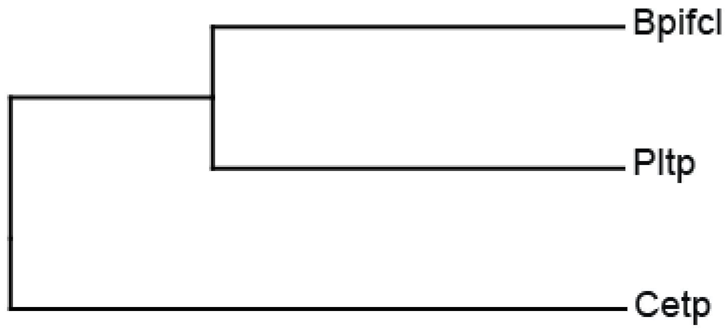

Supplementary Figure 1. Full-length mRNA and estimated amino acid sequences of *bpifcl* (A). Underline: N-terminal domain; dashed underline: C-terminal domain of the BPIF family predicted by SMART. Phylogenetic tree analysis of zebrafish BPIF family (B). Pltp (ENSDARG00000104495) and Cetp (ENSDARG00000030872) were used for this analysis.

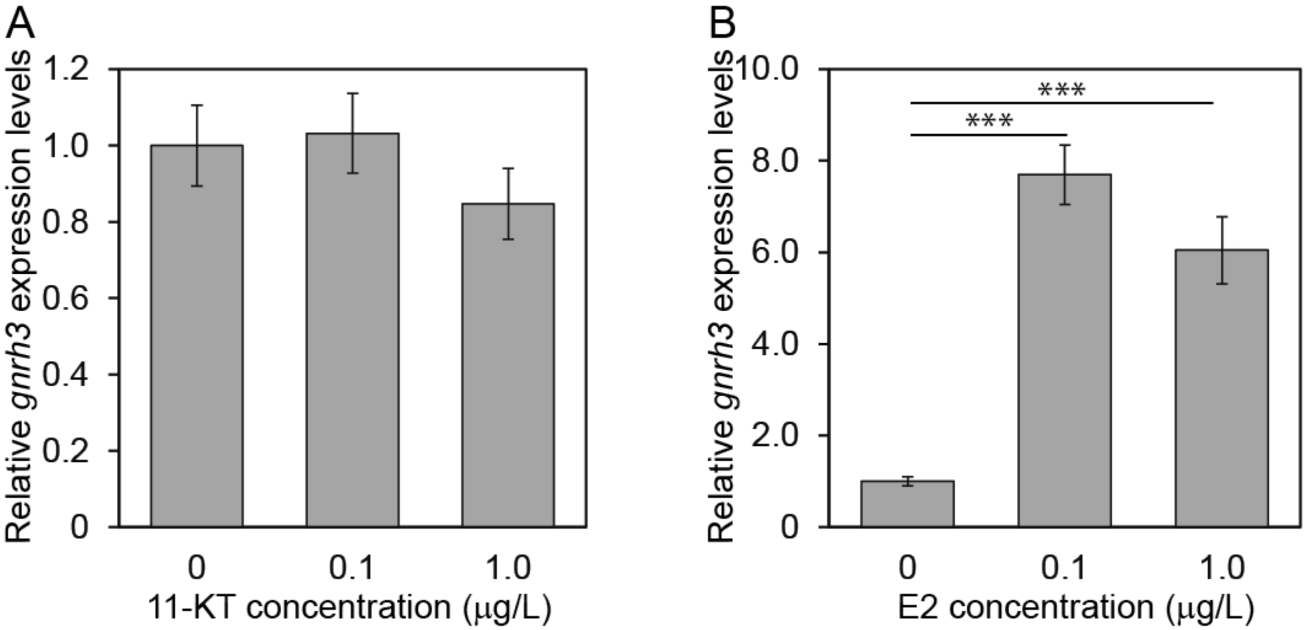

Supplementary Figure 2. Effect of 11-ketotestosterone (11-KT) exposure on *gnrh3* (A) gene expression in the female zebrafish brain. Effect of β-estradiol (E2) exposure on *gnrh3* (B) gene expressions in the male zebrafish brain. Expression levels in 0 μg/L were defined as 1.0. All of data are presented as mean±SEM and analyzed by a one-way ANOVA and Tukey's post hoc test for multiple comparisons. ***P<0.001.
